# Supplementary material for: Adiabatic topological photonic interfaces
Source: Nat Commun. 2023 Aug 2;14:4629. doi: 10.1038/s41467-023-40238-5 (PMC10397281; doi:10.1038/s41467-023-40238-5)
Supplement: Supplementary file 1 — Supplementary Information [file 41467_2023_40238_MOESM1_ESM.pdf]

Supplementary Information: Adiabatic photonic topological interfaces

Anton Vakulenko[1], Svetlana Kiriushechkina[1], Daria Smirnova[2], Sriram Guddala[1], Filipp Komissarenko[1], Andrea Alù[1,3], Monica Allen[4], Jeffery Allen[4], and Alexander B. Khanikaev[1,3*]✉

[1]Electrical Engineering and Physics, The City College of New York (USA), New York, NY 10031, USA
[2]ARC Centre of Excellence for Transformative Meta-Optical Systems (TMOS), Research School of Physics, The Australian National University, Canberra, ACT 2601, Australia
[3]Physics Program, Graduate Center of the City University of New York, New York, NY 10016, USA
[4]Air Force Research Laboratory, Munitions Directorate, Eglin AFB, USA.
These authors contributed equally: Anton Vakulenko, Svetlana Kiriushechkina
[*] corresponding author
✉email: khanikaev@gmail.com

This Supplementary Information includes:
Supplementary notes 1-5
Supplementary figures S1-S4
Supplementary references

Note 1: Analytical theory of the adiabatic topological interfaces

Neglecting spin-mixing and quadratic dispersion terms, which is good approximation in the close proximity to the Γ-point, the effective Dirac Hamiltonian for the patterned metasurface of hexamers[1-3] can be split into two independent, spin-up (↑) and spin-down (↓), blocks of the form:

$$\hat{H}_{\downarrow,\uparrow}(\boldsymbol{k}) = m\hat{\sigma}_z + v_D\left(\pm\tilde{k}_x\hat{\sigma}_x + \tilde{k}_y\hat{\sigma}_y\right). \quad \text{(S1)}$$

Adopting the plane-wave like ansatz for the two-component Dirac spinor $\boldsymbol{\psi}_{\uparrow/\downarrow} = [\psi_1, \psi_2]_{\uparrow/\downarrow}{}^{\mathrm{T}} \propto e^{-i\omega_{\uparrow/\downarrow}t + i\tilde{k}_y y}$ and replacing $\tilde{k}_x$ with a derivative $-i\partial_x$,we solve the eigenvalue problem

$$\omega_{\uparrow/\downarrow}\boldsymbol{\psi}_{\uparrow/\downarrow} = \hat{H}_{\uparrow/\downarrow}\boldsymbol{\psi}_{\uparrow/\downarrow}, \quad \text{(S2)}$$

with an antisymmetric adiabatic distribution of the mass term

$$m(x) = M_0 \tanh\left(\frac{x}{w}\right), \quad \text{(S3)}$$

where the inhomogeneity (width) parameter $w > 0$, and we assume $M_0 > 0$. Domain walls in the Dirac equation (S2) can host two types of modes - edge states and waveguide modes bound by the adiabatic interface. The spin-polarised edge modes, being associated with mass inversion at the domain wall, possess linear dispersion $\omega_{\uparrow/\downarrow} = \mp v_D\tilde{k}_y$, and spatial profiles obeying

$$\boldsymbol{\psi}_{\uparrow/\downarrow}(x) = \begin{pmatrix}\psi_1\\ \psi_2\end{pmatrix}_{\uparrow/\downarrow} = f(x)\begin{pmatrix}1\\ \mp i\end{pmatrix}, \quad \text{(S4)}$$

where

$$f(x) = \psi_1(0)\exp\left(-\frac{1}{v_D}\int_0^x m(x')dx'\right). \quad \text{(S5)}$$

The normalization factor for these modes is

$$\int_{-\infty}^{\infty}(|\psi_1(x)|^2 + |\psi_2(x)|^2)dx = 1 = 2\psi_1(0)^2\int_{-\infty}^{\infty}\exp\left(-\frac{2}{v_D}\int_0^x m(x')dx'\right)dx. \quad \text{(S6)}$$

On plugging (S3) into (S5), the mode profiles (S4) take the k_y-independent form

$$\begin{pmatrix}\psi_1\\ \psi_2\end{pmatrix}_{\uparrow/\downarrow} \propto \frac{1}{\cosh^{\kappa_0 w}\left(\frac{x}{w}\right)}\begin{pmatrix}1\\ \mp i\end{pmatrix}. \quad \text{(S7)}$$

For the step-like profile $m(x) = M_0\mathrm{sgn}(x)$ at $w \to 0$, solution (S4) is simplified to the exponentially confined function

$$\begin{pmatrix}\psi_1\\ \psi_2\end{pmatrix}_{\uparrow/\downarrow} = \sqrt{\frac{\kappa_0}{2}}\begin{pmatrix}1\\ \mp i\end{pmatrix}e^{-\kappa_0|x|}, \quad \text{(S8)}$$

with a constant decay rate $\kappa_0 = \frac{M_0}{v_D}$.

We employ the obtained profiles to estimate the quality factor of the electromagnetic modes in a realistic structure. The quality factor $Q = \mathrm{Re}(\omega)/(2\mathrm{Im}(\omega))$, which quantifies the radiative losses of leaky electromagnetic modes in the open system, is estimated as $Q = \omega_0 W/P$, where

$\omega_0 \equiv \mathrm{Re}(\omega)$ is the real part of the complex eigenfrequency, W is the stored energy of a mode, and P is the radiated power[4]. Following descriptions provided in[2,3], the radiative losses are associated with dipolar component of the field, which is given by $\psi_1(x)$. We can compute the local unit-cell-averaged dipole moment for the modes $\mathbf{p}(x)$: $p_x(x) = \pm i p_y(x) \propto \psi_1(x)$, and the corresponding surface polarization current distribution $\mathbf{J}(x,y) \propto -i\omega\mathbf{p}(x)e^{i\tilde{k}_y y}$ across the domain wall at some frequency ω:

$$J_{x,y}(x,y) \propto -i\omega e^{i\tilde{k}_y y} \sum_{m'=-\infty}^{+\infty} \psi_1(x)\delta(x - m'a) \propto e^{i\tilde{k}_y y} J_\delta(x)\,, \tag{S9}$$

where m' ennumerates the unit cells, a is a period of the lattice, and $J_\delta(x)$ is a discretised function following the spatial profile of the mode. The radiated power P in this phenomenological model for our subdiffractive thin metasurface is then calculated by Fourier transforming the 2D surface electric current distribution and integrating the out-of-plane (in z-direction) Poynting flux for partial plane waves over the wavenumber $\tilde{k}_x$. The amplitudes of the radiating plane-wave-like harmonics, $\propto \exp(i\tilde{k}_x x + i\tilde{k}_y y)$, in the surface current are proportional to the Fourier-transformed wave function

$$\tilde{J}(\tilde{k}_x) \propto \int_{-\infty}^{+\infty} \psi_1(x)\, e^{-i\tilde{k}_x x} dx. \tag{S10}$$

The out-of-plane Poynting flux is then obtained via integration

$$P(\tilde{k}_y) \propto \int_{-\sqrt{\tilde{k}_0^2-\tilde{k}_y^2}}^{\sqrt{\tilde{k}_0^2-\tilde{k}_y^2}} S_z(\tilde{k}_x, \tilde{k}_y)\, d\tilde{k}_x. \tag{S11}$$

of the partial contributions

$$S_z(\tilde{k}_x, \tilde{k}_y) \propto \left|\tilde{J}(\tilde{k}_x)\right|^2 \left(\frac{\tilde{k}_0}{\sqrt{\tilde{k}_0^2 - \tilde{k}_{||}^2}} + \frac{\sqrt{\tilde{k}_0^2 - \tilde{k}_{||}^2}}{\tilde{k}_0}\right), \tag{S12}$$

where $\tilde{k}_0$ is the free-space wavenumber, and $\tilde{k}_{||}^2 \equiv \tilde{k}_x^2 + \tilde{k}_y^2$ is the in-plane wavenumber. The estimate (S12) is done with the use of the interface boundary conditions for the magnetic field jump on the conducting plane. This description hints to the increase of the quality factor for the edge modes supported by smoother domain walls compared to their sharp counterparts due to narrowing the radiation spectrum, and agrees well with the results of our full-wave numerical simulations. The complex-valued spectrum calculated with the use of the Dirac model is illustrated by Fig. S1.

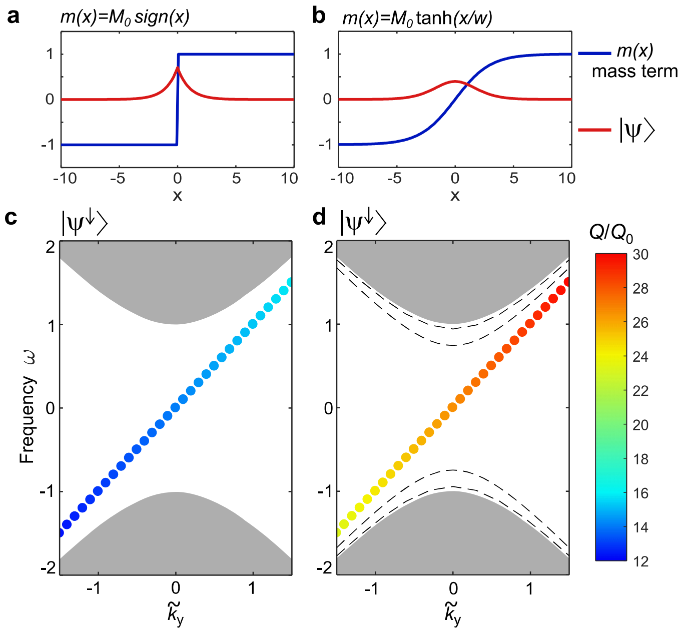

Fig. S1. Analytical model of the adiabatic topological interface. a-b, mass term profile $m(x)$ (blue line) and boundary mode field distribution ψ_1 (red line) for non-adiabatic step-like case (a) and adiabatic case (b). **c-d**, The complex spectrum calculated with the use of the Dirac model is shown for the pseudo-spin down ($\psi^{\downarrow}$) modes in non-adiabatic (a) and adiabatic case (b). The radiative quality factor of the modes is color-coded.

Similarly, for profiles demonstrated in the main manuscript we can obtain the mode localization function using (S5). For square root ($m(x) = \pm bx^{1/2}$) and linear ($m(x) = ax$) profiles of the mass term, the localization are not conventional exponential localization, but $\psi_{1,2}(x) \sim \exp\left\{-\frac{2}{3v_D} bx^{3/2}\right\}$ and a gaussian like $\psi_{1,2}(x) \sim \exp\left\{-\frac{1}{2v_D} ax^2\right\}$, respectively. Thus, modulation of the gauge field allows one to control field distribution in the direction perpendicular to the domain wall. Both simulations and experiment (Fig.1 and Fig.2) confirmed prediction that the field becomes less localized for smoother profiles, with the linear case yielding the least localized mode among tested profiles. This in turn gives rise to the better cancellations of the far-fields which are out-of-phase for the topological and trivial domain, as is evidenced by Fig. S2.

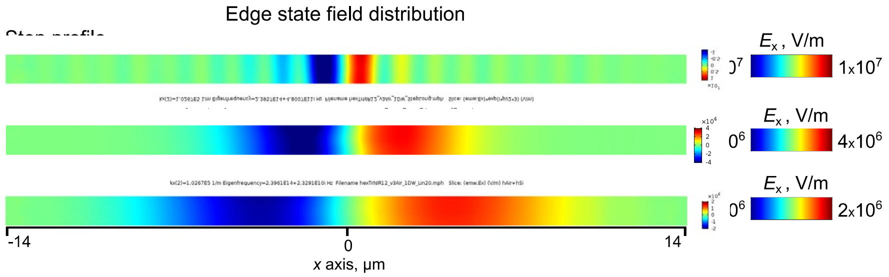

Figure S2. Simulation results for far field distributions of electrical field E_x of the edge mode for domain walls with different profiles. Field distribution is plotted in the *xy*-plane 1.5 μm above the metasurface.

Note 2: Comparison of sharp and adiabatic domain walls with the same usable bandwidth

In the main text we considered sharp and adiabatic profiles of domain walls with the same values of maximal degree of perturbation (shrinkage = 8%, expansion = 6%). Separated bulk guided modes in the case of adiabatic profile penetrate into the bandgap and thus decrease the effective bandwidth of the topological modes. In this note we provide first-principles simulation results for band structures of conventional step-like domain walls with lower degree of perturbation and the bandgap width matched to the one of adiabatic square-root and linear profiles (Fig. S3). Figure S2a shows results for the adiabatic square root profile and step profile with lower degree of perturbation (step2 and step3), both of which have a bandgap of approximately 68 nm. Figure S2b shows results for the linear profile and step profile with a bandgap of 41 nm width. While for narrower bandgap the quality factor of topological edge state for the step profile increases, yet it is several times lower than in the case of adiabatic profiles with the same bandgap. For example, at $|k_y/k_0| = 0.1$ calculations yielded $Q_{\text{sqrt}} = 1745$ and $Q_{\text{step2}} = 567$ (Fig.S2a), $Q_{\text{linear}} = 5890$ and $Q_{\text{step3}} = 2376$ (Fig.S2b).

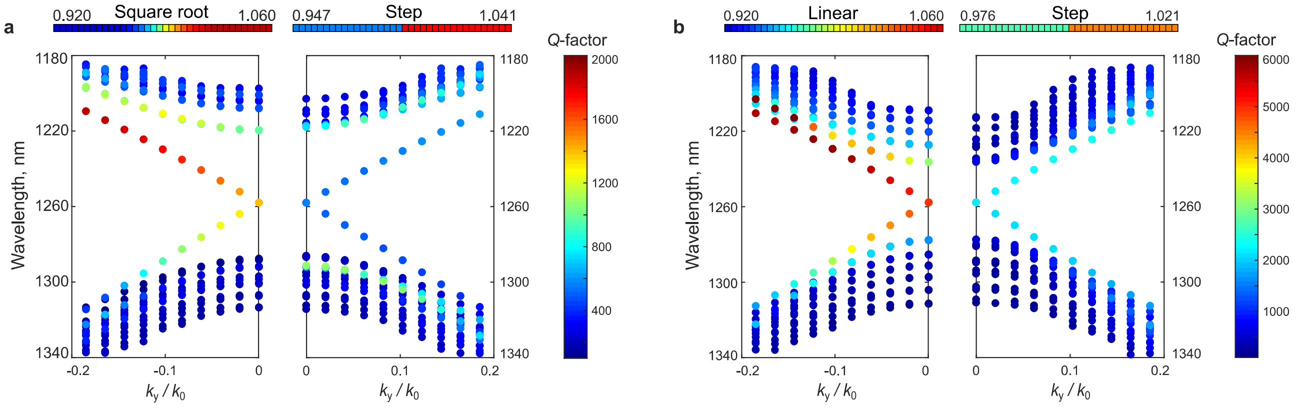

Figure S3. Comparison of domain walls with the step and adiabatic profiles with equivalent bandwidths. a,b Photonic band structure of topological boundary modes for adiabatic square-root mass-term profile and step profile with lower degree of perturbation in topological and trivial domains (**a**), and for adiabatic linear profile and abrupt step profile (**b**). Colors in the band diagrams show radiative quality factor of the edge modes and guided bulk modes. Upper insets show color-coded diagrams of the degree of perturbation of the mass-term profiles in the cross-section of the domain wall.

Note 3: Evolution of edge modes for adiabatic linear interface with increasing width

The boundary modes become progressively less localized when the domain wall transforms from abrupt to adiabatic kind of interface. As it was discussed in Note 1 for the specific case of linear profile, with the mass term $m(x) = ax$, the modes are not exponentially localized to the interface, but show the Gaussian profile $\psi(x,y) \sim \exp\left\{-\frac{1}{2v_D} a x^2\right\}$. Thus, for the case of infinitely wide linear region, $a \to \infty$, the modes become completely delocalized, i.e., they effectively represent

bulk modes. Additionally, some of the bulk modes start to localize to the linear transition region, and as its width approaches infinity, there is an infinite number of such modes, which continuously populate the former gap region (Fig. S4). Thus, the physics in the limit $a \rightarrow \infty$ becomes indistinguishable from the case of massless $m = 0$ and gapless Dirac equation. This behavior was confirmed by numerical modelling, as shown in Fig. S4, where the width of the linear interface was gradually changed from zero unit cells (step profile) to 37 unit cells in the direction perpendicular to the edge mode propagation direction.

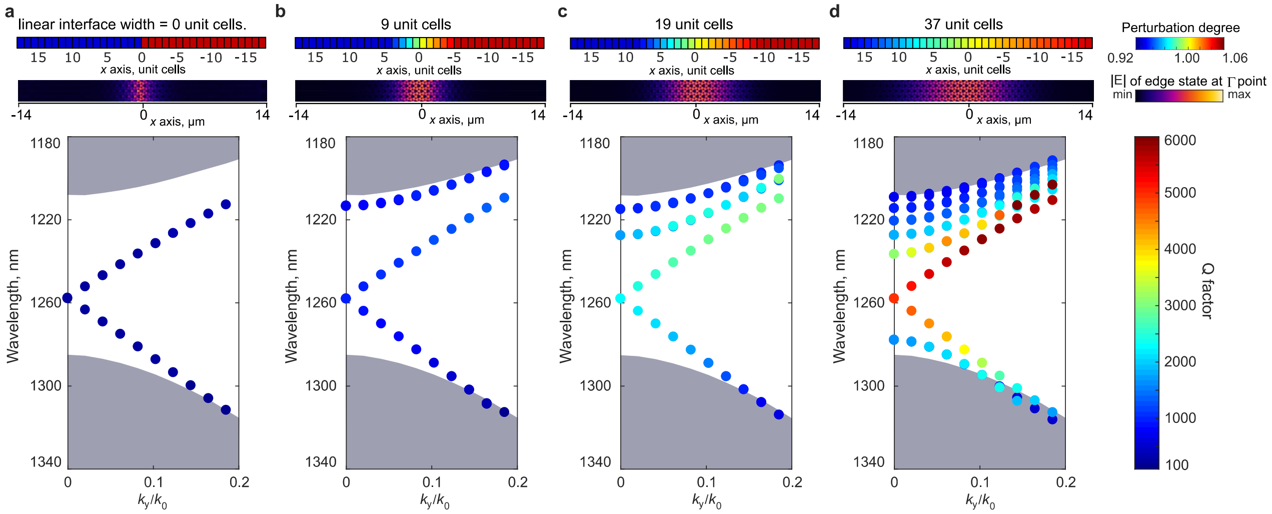

Figure S4. Comparison of adiabatic domain walls with increasing width. Photonic band structure of topological boundary modes for linear mass-term profile with gradually increasing width of the linear region (**a-d**). Color-coded diagrams of the degree of perturbation and edge mode near-field distribution for each interface are shown on the top panels. Radiative quality factors of the modes are indicated by color in these band diagrams.

To summarize, when considering the smoothness of the mass-term profile, one should consider tradeoffs and aim at the edge modes which are well-defined, i.e., exist within a reasonably wide topological bandgap, yet are sufficiently localized as needed for a particular application.

Note 4: Radiative coupling and energy stored in the edge modes

In experimental results it was noticeable that the radiative coupling of edge states is getting smaller for smoother profiles, it is becoming harder to couple incident light into the modes from the far field. Thus, one might expect less energy in the edge states for smoother profiles. However, the reduction in coupling efficiency is also partially compensated by the increased quality factors of the modes. The simplest description of these two competing mechanisms can be made in the framework of the coupled mode theory[5], which allows one to estimate the mode amplitude as:

$$A = \frac{i\alpha}{\omega - \omega_0 + \frac{i}{\tau}}, \tag{S13}$$

where α is the radiative coupling efficiency of the mode, which is related to the radiative lifetime τ_R as $\alpha = \sqrt{1/\tau_R}$, $\tau = (\tau_R^{-1} + \tau_0^{-1})^{-1}$ is the total lifetime of the edge mode, and τ_0 is the

lifetime of the mode due to all other processes, including absorption and scattering. Assuming resonant excitation, we see that the energy stored in the mode is given by:

$$W \sim |A|^2 = \tau_R \tau_0^2 / (\tau_R + \tau_0)^2 \ . \tag{S14}$$

In the limit of very large τ_R this yields $W \sim \tau_0^2/\tau_R$, i.e., the energy captured by the mode from the incident radiation will drop inverse proportionally to the radiative lifetime of the mode. At the same time, the reflectance drops even faster $R = W/\tau_R \sim \tau_0^2/\tau_R^2$. Thus, although we might see very little power outflow from the edge mode (resulting in lower signal-to-noise ratio), there is still a significant amount of energy stored in the mode.

We note, however, that the coupling from the far field is not the only way to excite boundary modes, and for very high radiative quality factors (as well as for most practical purposes), one would rather use coupling via grating integrated into the structure.

Note 5: Boundary modes with symmetry-reducing defect

We have performed numerical modelling and analyzed the effect of a local (symmetry-reducing) defect on the boundary modes. Introduction of such the defect is known to give rise to the pseudo-spin flipping, coupling between oppositely propagating edge states, and, eventually, gapping of the boundary modes. Thus, to understand the effect of the defect, we have calculated bandwidth of the gap between oppositely propagating modes for mass term profiles of different adiabaticity. We found that, indeed, due to the more spread field distributions for smoother profiles, edge modes were increasingly less sensitive to the defect, and the size of the defect-induced gap was respectively smaller. The width of the gap was calculated as the function of the symmetry reduction strength for three profiles of interest, showcasing the strongest robustness of the least localized linear mass term profile (Fig. S5).

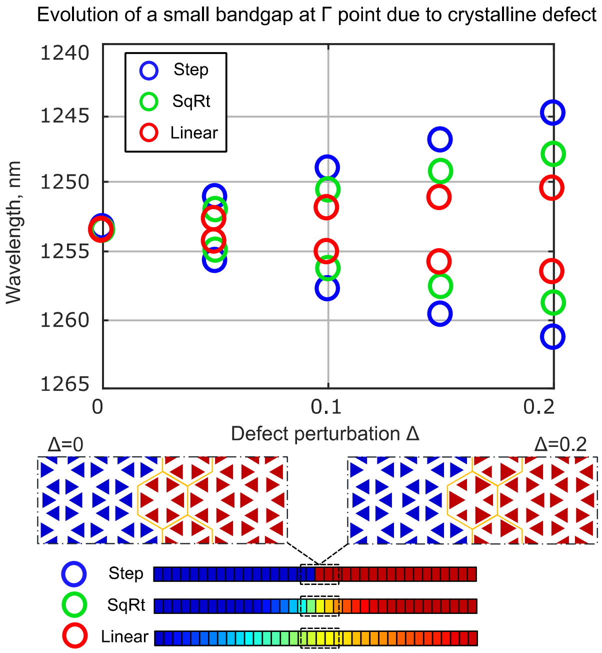

Figure S5. Simulations results for bandwidth of the defect induced gap at the Γ point for different mass term profiles for increasing local crystalline defect perturbation. Defect perturbation Δ and its location at the interface (white rectangle) are shown alongside the mass term profiles in lower panel. The defect represents the symmetry reduction at one unit cell adjacent to the domain wall within the supercell. The average dielectric constant of the defective unit cell was kept the same (the volume fraction of air holes is maintained), which ensures that the center of the gap remain at the same spectral position.

Supplementary references:

1. Barik, S. *et al.* A topological quantum optics interface. *Science.* **359**, 666–668 (2018).
2. Gorlach, M. A. *et al.* Far-field probing of leaky topological states in all-dielectric metasurfaces. *Nat. Commun.* **9**, 909 (2018).
3. Smirnova, D. *et al.* Third-Harmonic Generation in Photonic Topological Metasurfaces. *Phys. Rev. Lett.* **123**, 103901 (2019).
4. Vučković, J., Lončar, M., Mabuchi, H. & Scherer, A. Optimization of the Q factor in photonic crystal microcavities. *IEEE J. Quantum Electron.* **38**, 850–856 (2002).
5. Haus, H. A. *Waves and fields in optoelectronics*. *Ch. 7* (Englewood Cliffs, NJ: Prentice-Hall, 1984).
